# Impacts of Short-Term Grazing Intensity on the Plant Diversity and Ecosystem Function of Alpine Steppe on the Qinghai–Tibetan Plateau

**DOI:** 10.3390/plants11141889

**Published:** 2022-07-21

**Authors:** Xinghai Hao, Juejie Yang, Shikui Dong, Hao Shen, Fengcai He, Yangliu Zhi, Emmanuella A. Kwaku, Danjia Tu, Shengyun Dou, Xueli Zhou, Zhengrong Yang

**Affiliations:** 1 School of Grassland Science, Beijing Forestry University, Beijing 100083, China; hxh199801@163.com (X.H.); shenhao2222@gmail.com (H.S.); fengcai_he0327@163.com (F.H.); 2 State Key Joint Laboratory of Environmental Simulation and Pollution Control, School of Environment, Beijing Normal University, Beijing 100875, China; yangliu_zhi@163.com (Y.Z.); emmkwaku.17@gmail.com (E.A.K.); 3 Grassland Improvement Experimental Station of Qinghai Province, Gonghe 813099, China; tdjqh2022@163.com (D.T.); tiebujiacaoye@126.com (S.D.); zhouxuelia@163.com (X.Z.); 18609716488@163.com (Z.Y.)

**Keywords:** grazing intensity, species composition, plant diversity, ecosystem multifunctionality, alpine steppe

## Abstract

Livestock grazing is the primary land use of grasslands worldwide. Grazing has been asserted to alter grassland ecosystem functions, such as productivity, nutrient cycling, and biodiversity conservation. However, few studies have focused on the impact of grazing intensity on the ecosystem multifunctionality (EMF) of alpine grasslands. We conducted a field experiment of manipulating sheep grazing intensity effects on alpine steppe by surveying plant community characteristics and ecosystem functions. Our results showed that plant community composition was altered with increasing grazing intensity, and the dominant species shifted from grasses and sedges to forbs. EMF was the highest under no grazing (CK) and the lowest under heavy grazing (HG), but there was insignificant difference between CK and HG. HG significantly decreased some indicators that reflected nutrient cycling functions, such as soil available nitrogen, plant leaf nitrogen (PN) and phosphorus content (PP). Furthermore, plant diversity had strong correlations with SOC, total nitrogen (TN), and PN. The results could provide scientific bases for biodiversity conservation and sustainable grazing management of alpine steppe.

## 1. Introduction

The Qinghai–Tibetan Plateau (QTP), well-known as the “Water Tower” of Asia and the “the third pole” of the world, covers about 26% of the terrestrial land area in China [1]. The dominant ecosystems of QTP are alpine grasslands (mainly alpine meadow and alpine steppe), which are principally used for yak and Tibetan sheep grazing [2]. As the main body of the QTP ecosystem, alpine grassland is one of the areas with the richest and most concentrated species and genetic genes in the alpine ecosystem, which plays a very important role in the global alpine biodiversity protection [3]. In addition to providing livestock products, alpine grasslands also provide vital ecosystem functions such as productivity, nutrient cycling, and resource acquisition [4]. However, under the dual pressures of human activities and global climate change, alpine grasslands are currently experiencing high rates of degradation, which may threaten grassland biodiversity and ecosystem structure and functioning [5].

Livestock grazing is the primary land use and human disturbance of grassland worldwide. Grazing by herbivores affects plant communities and ecosystem functions through a variety of mechanisms, including trampling, selective feeding, and fecal excretion of livestock [6,7,8]. Grassland plant diversity and ecosystem functions have different responses to grazing intensity. HG may have a negative effect on plant species diversity and simplify the structure of plant communities [9]. Moderate grazing (MG) may enhance the diversity of plant species in the grasslands by stimulating the photosynthesis capacity of plants [10]. According to the optimal partitioning theory, plants allocate more resources to sustain belowground root growth under MG [11]. Light grazing (LG) and MG could promote the aboveground net primary productivity because of plant growth compensation mechanisms [12]. Livestock trampling and fecal input affect the soil properties, microbial community structure, and nutrient cycling [13,14,15]. Grazing could change the aboveground and underground biomass, and could then affect the input of soil C and N [16]. With different grazing intensity, grassland soil C and N pools might increase or decrease [17]. Zhan et al. [11] showed that LG could improve the sequestration of soil C, while HG led to C being lost from soils across China’s grassland ecosystems. A meta-analysis about the grazing effects on QTP grasslands indicated that TN was significantly reduced by 23.7% under HG [18]. Moreover, Dao et al. [19] demonstrated that MG could improve the availability of plant nitrogen and phosphorus.

Over the past few decades, a large number of studies have focused on the optimal grazing management strategy for the maintenance of single ecosystem function, including productivity, species diversity, and soil carbon storage [20,21,22]. However, ecosystem functioning is inherently multiple, and multifunctionality can be used to summarize the ability of an ecosystem to provide multiple ecosystem functions simultaneously [23]. Recently, there has been increasing concern about the effects of grazing on grassland EMF. Assessing how grazing intensity impact grassland EMF is crucial for better grazing management practices. Zhang et al. [24] found that EMF decreased with increasing grazing intensity in desert steppe ecosystems, and plant diversity played a key role in regulating the response of EMF to grazing intensity. Wang et al. [25] considered 12 ecosystem functional variables related to the cycling of C, N, and P and productivity to assess EMF, and they found that MG could promote grassland biodiversity and EMF in a steppe grassland. As one of the world’s most fragile ecosystems, alpine grasslands are more sensitive than other grasslands in response to grazing disturbance [26]. However, the responses of plant characteristics and EMF in alpine grassland to different grazing intensities are still unclear.

In this study, we conducted a grazing experiment with four intensities, including CK, LG, MG, and HG, to evaluate the effects of grazing intensity on plant species composition, plant diversity, and the EMF of the alpine steppe on QTP. Eight ecosystem functional indicators, i.e., aboveground net primary productivity (ANPP), belowground biomass (BGB), soil organic carbon (SOC), soil total nitrogen (TN), soil available phosphorus (AP), soil available nitrogen (AN), plant leaf nitrogen content (PN), and plant leaf phosphorus content (PP), which are related to nutrient cycling and primary production, were chosen to assess EMF. The relationships between plant diversity and EMF under different grazing intensities were also evaluated. We hypothesized the following: (1) plant community composition is altered with increasing the grazing intensity and forbs would become the dominant species under HG, (2) EMF would decrease with increasing the grazing intensity in the alpine steppe, (3) and there is a strong relationship between plant diversity and soil functions. The results of this study could provide bases for bettering grazing management of QTP’s alpine steppes in order to conserve plant diversity and sustain ecosystem functions.

## 2. Results

### 2.1. Effects of Grazing Intensity on Plant Species Composition of Alpine Steppe

**Figure 1:**
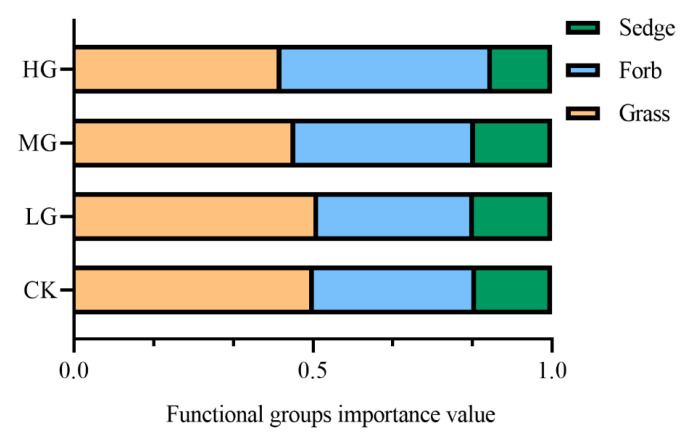
The importance values of functional groups under different grazing intensities. CK, no grazing; LG, light grazing; MG, moderate grazing; HG, heavy grazing.

The species composition of alpine steppe communities changed with increasing the grazing intensity. Grazing decreased the importance value (IV) of grass species such as *Leymus secalinus* (Georgi) Tzvelev. HG had negative effects on the IV of the sedge species, *Carex parvula* O. Yano (Table 1). The IVs of grasses and sedges reached the maximum under LG (Figure 1). With increasing the grazing intensity, forb species such as *Potentilla multifida* L., *Gentiana macrophylla* Pall., *Irisensata* thumb., and *Thermopsis lupinoides* (L.) Link appeared in the community. Compared with CK, the IVs of forbs significantly (*p* < 0.01) increased by 30.25% under HG (Figure 1). Forbs tended to dominate the plant communities under HG.

### 2.2. Effects of Grazing Intensity on Plant Species Diversity of Alpine Steppe

The MG plots had the highest Margalef richness index (*Dmg*) among all of the plots, but no significant differences were observed among the different grazing intensity plots (*p* > 0.05). In addition, there were no significant differences in the Shannon–Wiener diversity (*H*’), Simpson (*D*), and Pielou (*J*) indexes among all of the grazed grassland with different grazing intensities (*p* > 0.05), and all three were the highest in the HG grassland (Table 2).

**Figure 2:**
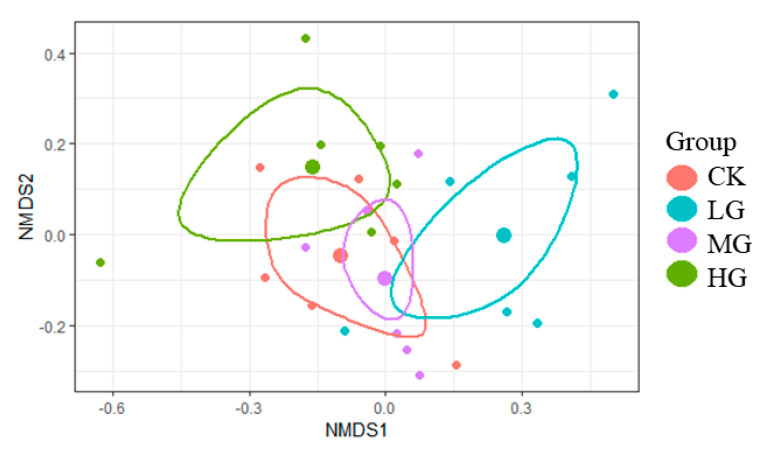
NMDS analysis of the plant community based on the data of the species importance value (IV) with different grazing intensities. Dots of the same color represent the same level of grazing intensity. Each small dot indicates a treatments replication, and each large dot shows the mean value of the treatments. Circles represent the 95% confidence of the mean value. CK, no grazing; LG, light grazing; MG, moderate grazing; HG, heavy grazing.

The NMDS analysis demonstrated that the plant communities of CK, LG, and HG grasslands were clustered in different patterns. The plant communities between CK and MG had a high similarity (Figure 2). PER-MANOVA analysis further proved the significant difference (*p* < 0.05) between CK and LG. Significant differences in the plant communities were also observed between LG and HG (*p* < 0.01).

### 2.3. Effects of Grazing Intensity on Ecosystem Function Indicators and EMF

The dynamics of eight ecosystem function indicators are presented in Table 3. The results show that LG had the highest ANPP (633.07 g m^−2^), while CK had the lowest ANPP (323.63 g m^−2^). There were no significant changes of ANPP among the different grazing intensities (*p* > 0.05). Grazing intensity had insignificant effects on BGB, while the highest BGB was found under MG. Grazing intensity had significant effects on AN, PN, and PP (*p* < 0.05), but it had insignificant effects on SOC, TN, and AP (*p* > 0.05). Compared with CK, LG and HG significantly reduced the soil AN by 40.62% and 34.40%, respectively (*p* < 0.05). The highest PN (19.96 mg kg^−1^) and PP (1.37 mg kg^−1^) were observed under LG. Compared with CK, the LG significantly (*p* < 0.05) reduced PN by 20.11%. Compared with LG, the HG significantly (*p* < 0.05) reduced PN and PP by 24.75% and 21.90%, respectively.

**Figure 3:**
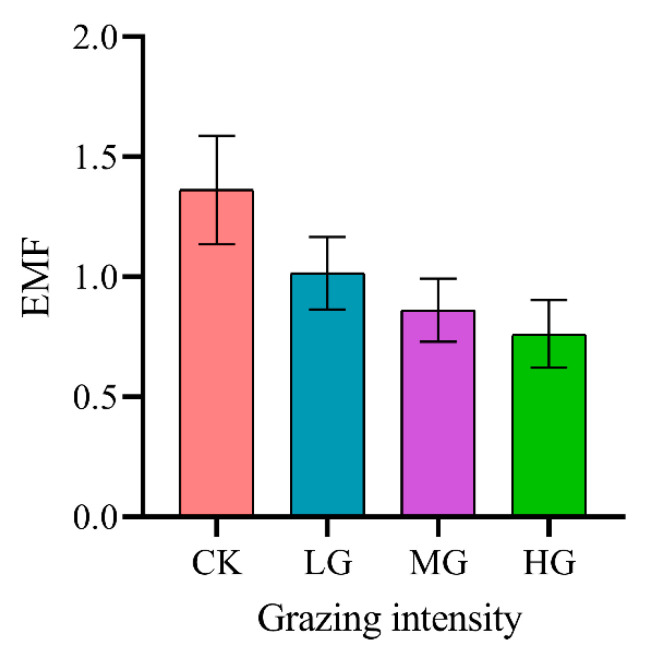
The effect of grazing intensity on ecosystem multifunctionality (EMF). Different lowercase letters show significant differences between means (*p* < 0.05). The non-significant differences are not marked by any letters. CK, no grazing; LG, light grazing; MG, moderate grazing; HG, heavy grazing.

The EMF calculated from the eight ecosystem function indicators are presented in Figure 3. The results show that CK had the highest EMF and HG had the lowest EMF among the grazing treatments. Grazing intensity had an insignificant (*p* > 0.05) impact on EMF, but there was a gradually decreasing trend with the increasing grazing intensity. Compared with CK, LG, MG, and HG decreased the EMF by 25.48%, 36.76%, and 44.10%, respectively.

### 2.4. Relationships between Plant Diversity and Ecosystem Function Indicators and EMF under Grazing

**Figure 4:**
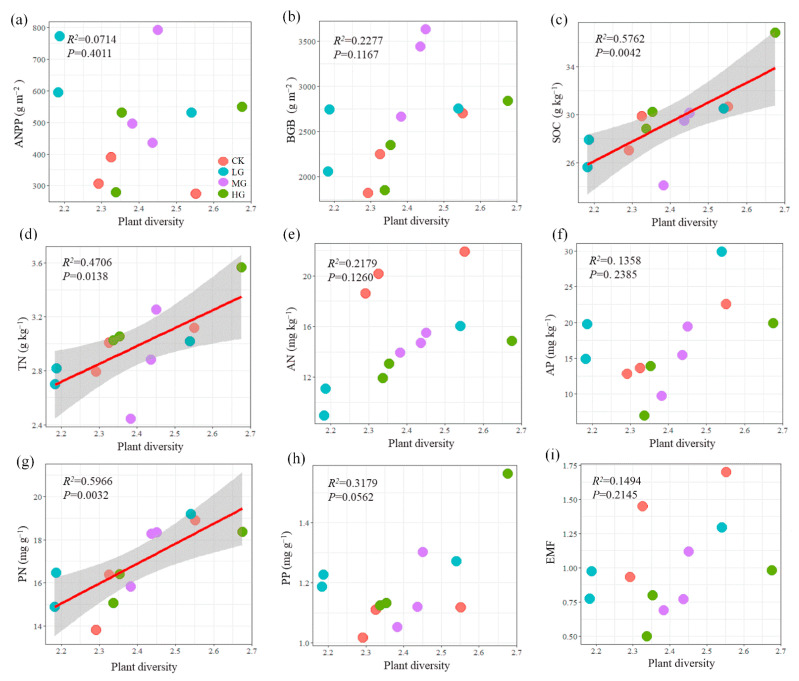
Relationships between plant diversity and ecosystem function indicators (**a**–**h**) and ecosystem multifunctionality (EMF) (**i**) under grazing. The solid lines indicate statistically significant relationships (*p* < 0.05). Shaded areas show the 95% confidence interval of the fit.

The relationships between plant diversity (Shannon–Weiner index) and the eight ecosystem function indicators are presented in Figure 4. There was a significant (*p* < 0.05) positive linear relationship between plant diversity and SOC, TN, and PN under all of the grazing intensities (Figure 4c,d,g). Insignificant relationships were detected between plant diversity and other function variables (Figure 4a,b,e,f,h). ANPP, BGB, AN, and LP showed an increasing trend with increasing the plant diversity (*p* > 0.05). In contrast, negative linear relationships were observed between plant diversity and AP. EMF had an insignificant (*p* > 0.05) relationship with plant diversity (Figure 4i).

## 3. Discussion

### 3.1. Effect of Grazing Intensity on Community Characteristics of Alpine Steppe

Our results indicate that HG reduced the proportion of grasses and sedges and increased the forbs (Figure 2), which was consistent with other studies [27,28]. This was probably because selective browsing by herbivores influenced competitive relationships between plant species [29]. Because of the specific feeding preference of the sheep, the livestock preferentially chose grasses [30]. The proportion of grasses decreased with the increase in grazing intensity. Grazing reduced the competitive ability of grasses in the upper layer of the community, which provided more resources for the growth of forbs [31]. Therefore, forbs had a greater competitive ability than grasses, and the proportion of forbs increased under HG [32].

Plant diversity was a measurable indicator reflecting the community structure, which has various responses to grazing intensity [33]. Numerous studies have shown that moderate grazing increased plant diversity, known as the “intermediate disturbance hypothesis” [34]. Our results found insignificant differences in the Shannon–Wiener (*H*’), Simpson index (*D*), Pielou (*J*), and Margalef index (*Dmg*) among the different grazing intensities. Inconsistent with our results, previous findings have suggested that grazing disturbance reduced the plant diversity, resulting in a single community structure [35]. It was possible that the duration of grazing experiments, the type of livestock, and the ecosystem types led to different effects of grazing on the species diversity [36,37]. In this study, the responses of diversity indexes to grazing were not sensitive, but the Shannon–Wiener diversity (*H*’) and Pielou evenness (*J*) were the highest under HG. The selective browsing by livestock inhibited the growth of dominant species, which made it possible for the invasion and settlement of unpalatable plant species [38]. Forbs had favorable conditions (resources and space) for the growth and development, and thus increased the complexity of the community structure and plant diversity under HG [39]. Moreover, the NMDS analysis and further PER-MANOVA analysis demonstrated that the plant community composition was altered with increasing the grazing intensity. Similarly, Song et al. [40] found that the plant communities were obviously different between the no grazing plots and the grazing plots.

### 3.2. Effect of Grazing Intensity on Ecosystem Functions

Our results showed that grazing intensities had no significant effects on EMF, and HG maintained low levels of EMF. Consistently, HG significantly reduced most separate ecosystem functions and EMF in a 12-year grazing experiment of desert steppe [24]. Wang et al. [25] showed that MG maintained high levels of EMF and enhanced ecosystem functions (nutrient cycling and plant productivity) in a semi-arid steppe grassland. In the present study, only several ecosystem function indicators decreased significantly under LG and MG. Our results revealed that HG significantly reduced the soil AN. This was consistent with the finding that HG induced the loss of soil AN in a meta-analysis about the grazing effects in global grassland ecosystems [17]. This result may be attributed to the fact that trampling led to soil compaction and increased soil bulk density under HG, which was suitable for the survival of denitrifying bacteria [41]. Denitrification was enhanced, resulting in the loss of AN. Grazing altered the nutrient utilization of plants via trampling and inputting livestock excretion, and the response of the leaf nutrient content to grazing intensities varied [42]. Our results demonstrated that HG significantly decreased the PP and PN content. A possible explanation was that HG led to the loss of soil AN, and the amount of nitrogen absorbed by plants decreased accordingly [43]. Moreover, nitrogen and phosphorus in plants are synergistic elements, generally showing a positive correlation, so the PP content decreased accordingly [44].

Previous studies have shown that the plant compensatory growth occurred in alpine ecosystems, which was beneficial for enhanced plant growth under an appropriate grazing intensity [45,46]. In this study, although ANPP was not significantly changed under grazing, the compensatory growth of plants may be the reason for the highest ANPP of the community under LG. BGB was an important part of the productivity of the community, and played a key role in supporting belowground functionality [47]. Our results showed that the highest BGB was observed under MG. This can also be likely attributed to grazing induced plant compensatory growth, leading to the increase of BGB under MG [46]. In addition, plants reduced the AGB and allocated more resources to support the belowground root growth under MG for adapting to the environmental stress [11].

In our study, there were no significant changes in the soil ecosystem function indicators (SOC, TN, and AP). This might be attributed to the alpine grassland, which was characterized by low temperatures and low oxygen concentrations at high altitudes [48]. In alpine regions, the grassland plants had a short growing season and the grassland soil microbial activities were low [26]. The results obtained in this study indicated that HG increased the SOC and TN content in alpine grassland ecosystems. Consistently, previous studies have also reported that HG promoted increases in SOC and TN in the alpine grassland [14]. The fragile alpine environment and soil compaction due to trampling affected the activities of microbials, limiting the soil nutrients decomposition in the alpine grassland [26]. Our results indicated that grazing intensity showed no apparent effects on the soil AP content. The soil AP were relatively stable under different grazing intensities in the alpine steppe [49,50]. Overall, EMF showed a decreasing trend with the increasing grazing intensity. We suggest that grazing with low grazing pressure may improve the EMF of grassland ecosystems. Enclosure was the effective management mode in the short-term for the sustainable utilization of alpine grassland, considering that alpine steppes are currently experiencing degradation.

### 3.3. Relationships between Plant Diversity and Ecosystem Functions

A number of studies have demonstrated that plant diversity tends to be positively related to ecosystem function [51,52]. Wang et al. [25] reported that plant diversity was related to the ecosystem functions of C and N cycling in a typical steppe. Chen et al. [52] reported that plant diversity enhances productivity and soil carbon sequestration. Consistently, we found that plant diversity had strong correlations with soil function indicators (SOC and TN) under all of the grazing treatments. Plant diversity played a critical role in maintaining the key ecosystem processes, such as improving soil carbon sequestration and soil fertility [53]. Plant diversity promoted soil functioning through the facilitation relationship between plant species in the community and complementary resource use. Wang et al. [47] stressed that plant diversity had strong correlations with EMF in managed grasslands, owing to the niche complementarity effects of the plant community. Similar positive relationships between plant diversity and EMF were found globally across drylands [54]. However, plant diversity was not related to EMF in this study, likely due to the competition between the plant species and the mapping relationship between plant species and ecosystem functions [55,56]. As discussed above, our experiment adopted a relatively small number of plots to test the relationship of diversity function under different grazing intensities and the short duration of grazing experiments at present. So, the grazing effects in our study were short-term. Therefore, in future research, (a) long-term observations about plant diversity and ecosystem functions under different grazing intensities are required to further improve our understanding of the grazing effects in alpine steppe; (b) sampling methods should be focus on eliminating experiment deviation, and spatial heterogeneity; and (c) individual species biomass and biomass of functional groups (e.g., grasses, legumes, and forbs) should be monitored under different grazing intensities to explain the existence of compensatory species responses.

## 4. Materials and Methods

### 4.1. Study Sites

The grazing experiment was conducted in an alpine steppe located in Tiebujia Town, Qinghai Province, China (37°02′ N, 99°35′ E, 3270 m). The annual average precipitation, evaporation, and temperature are 377 mm, 1484 mm, and 0 °C, respectively. The vegetation is dominated by *Leymus secalinus*., *Poa pratensis*., *Koeleria cristata*., and *Aster tataricus*. The region has a typical plateau continental climate and the soil type is loam–clay soil. The experiment was established in 2019, and the vegetation and soil sampling for analysis in this study was in mid-August 2020.

### 4.2. Experimental Design

Before the research was carried out, all experimental plots were enclosed grasslands, which had the same basic conditions as the control treatment at the study site. The experiment was initiated in 2019 and four treatments (each treatment with three replicates) were designed using a completely randomized block in this study (a total of 12 plots). In accordance with the carrying capacity investigations by the local department in alpine steppe, we combined a previous study [57] and the current local grazing conditions. Ultimately, we identified the four stocking rate levels: no grazing 0 sheep hm^−2^ (CK), light grazing with 4 sheep hm^−2^ (LG), moderate grazing with 6 sheep hm^−2^ (MG), and heavy grazing 8 sheep hm^−2^ (HG). The size of each plot was 0.5 hm^2^, and grazing started from June to early September every year.

### 4.3. Vegetation and Soil Sampling

Vegetation and soil sampling were conducted in mid-August 2020 in this study. We randomly located two 1 m × 1 m quadrats in each plot to survey the plant species composition, species abundance, species diversity, and plant cover. Aboveground biomass (AGB) was harvested and weighed after 24 h of oven-drying at 65 °C. The aboveground leaf dry material was ground to a fine powder using a ball mill, and then measured for the PP and PN content. We determined PN using an elemental analyzer (EuroEA 3000, Pavia, Italy). The PP content was measured using inductively coupled plasma spectrometers (ICP) (SPECTRO ARCOS EOP, Kleve, Germany).

For the CK plots, the total aboveground biomass was treated as ANPP. For the grazing plots, we randomly located 1 m^3^ iron cages before each grazing in each plot, and then calculated the livestock feed intake by the difference in the dry matter content of the forage between inside and outside the cage. Therefore, ANPP was record as the sum of the biomass of the residual plants after grazing and the sum of feed intake during grazing. We estimated BGB by randomly collecting soil cores from two quadrats in each plot (0–20 cm depth, 7 cm diameter). We then collected the living root biomass by rinsing the soil samples in water using sieves (mesh size 0.25 mm), which were then oven dried at 65 °C for 48 h and weighed.

Soil samples were randomly collected from three soil cores (3.5 cm-diameter) at a depth of 20 cm in each plot, which was mixed to produce a composite sample. The samples were sieved through a 2 mm mesh, and then used to determine the soil parameters. Soil TN was measured using an elemental analyzer (EuroEA 3000, Pavia, Italy). We determined the soil AP using inductively coupled plasma spectrometers (ICP) (SPECTRO ARCOS EOP, Kleve, Germany). The soil AN (soil available NO_3_^−^ and NH_4_^+^) were determined using a FIAstar 5000 Analyzer. We determined the SOC content with the K_2_Cr_2_O_7_ titration method after digestion.

### 4.4. Statistical Analysis

#### 4.4.1. Species Importance Value and Species Diversity Index

The importance value (IV) of each plant species was calculated by using the following formula:

IV = (relative cover + relative height + relative frequency)/3(1)

In addiition, we calculated the Margalef richness index (*Dmg*), Shannon–Wiener diversity index (*H*′), Pielou evenness index (*J*), and Simpson dominance index (*D*) using the following formula [58,59,60,61]:

(2) $$H^{\prime }=-\sum_{i=1}^{s}P_{i}\ln P_{i}$$

(3) $$J=H^{\prime }/\ln S$$

(4) $$D=1-\sum_{i=1}^{s}P_{i}^{2}$$

(5) $$D_{\mathrm{mg}}=(S-1)/\ln S$$

where *P_i_* is the importance value of each plant species, *S* is the number of species, and *N* is the total number of individuals in the community.

#### 4.4.2. Assessing Ecosystem Multifunctionality

In this study, we selected eight ecosystem functions, namely, ANPP, BGB, SOC, TN, AP, AN, PN, and PP (Table 4). All individual ecosystem functions together reflect EMF. Then, we calculated the Z-scores of eight ecosystem functional variables and averaged them to calculate the EMF index [24]. The information on all ecosystem function indicators and their importance are listed in Table 4.

#### 4.4.3. Statistical Analysis

Nonmetric multidimensional scaling (NMDS) was performed using the “vegan” package in R (4.1.3) (Robert Gentleman and Ross Ihaka, Auckland, New Zealand) to examine species composition among different grazing intensities. Pairwise comparison for the PER-MANOVA test in R (Robert Gentleman and Ross Ihaka, Auckland, New Zealand) further proved whether there was a significant difference between the groups. One-way analysis of variance (ANOVA) was conducted to compare the differences in the diversity index using eight ecosystem function indicators. We used Tukey’s multiple comparisons to test for significant differences among all treatments (*p* < 0.05). ANOVA analysis was performed in SPSS 26.0 (Norman H. Nie, C. Hadlai (Tex) Hull, and Dale H. Bent, Chinago, IL, USA). We also checked the relationships of plant diversity (Shannon–Wiener diversity index) with ecosystem functions and EMF using general linear models (GLMs), namely “ggplot2” packages in R (Robert Gentleman and Ross Ihaka, Auckland, New Zealand), and GraphPad Prism 8.0 (Harvey Motulsky, San Diego, CA, USA) was used for drawing.

## 5. Conclusions

Our results demonstrated that grazing intensity greatly affected the plant diversity and ecosystem function of the alpine steppe. The dominant species in the plant community shifted from grasses and sedges to forbs with increasing grazing intensity. The Shannon–Wiener diversity and Pielou evenness were highest under HG. Compared with CK, HG significantly decreased the soil AN, PP, and PN by 34.40%, 24.75%, and 21.90%, respectively. Grazing intensities had no significant effects on most ecosystem function indicators and EMF, but HG maintained low levels of EMF. In addition, we found that plant diversity was positively correlated with SOC, TN, and PN under grazing. We suggest low grazing pressure for the QTP’s alpine steppe to maintain plant diversity and sustain the ecosystem functions.

## Figures and Tables

**Table 1:** Importance value indexes (IV) of species under different grazing intensities.

Family	Species	CK	LG	MG	HG
*Poaceae*	*Leymus secalinus* (Georgi) Tzvelev.	0.207	0.128	0.168	0.179
	*Poa pratensis* L.	0.092	0.081	0.079	0.085
	*Agropyron cristatum* (L.) Gaertn.	0.049	0.067	0.048	0.048
	*koeleria cristata* (Linn.) Pers.	0.072	0.144	0.099	0.059
	*Stipa capillata* L.	0.083	0.088	0.068	0.054
	*Elymus nutans* Griseb.	—	—	—	0.008
*Cyperaceae*	*Carex parvula* O. Yano	0.151	0.179	0.161	0.125
	*Carex melanantha* C. A. Mey.	0.008	—	—	—
*Fabaceae*	*Thermopsis lupinoides* (L.) Link	0.009	—	—	0.007
	*Medicago ruthenica* (L.) Trautv.	0.002	—	—	0.005
	*Astragalus membranaceus* (Fisch.) Bunge.	0.008	0.010	0.041	0.019
*Rosaceae*	*Potentilla bifurca* L.	0.032	—	0.008	0.028
	*Sibbaldia adpressa* Bge.	0.011	—	—	0.006
	*Potentilla multifida* L.	0.022	0.017	0.039	0.089
*Compositae*	*Aster tataricus* L. f.	0.077	0.117	0.081	0.086
	*Taraxacum mongolicum* Hand.-Mazz.	0.031	0.005	0.008	0.010
	*Artemisia frigida* Willd.	0.020	0.021	0.007	0.011
	*Artemisia scoparia* Waldst. et Kit.	0.027	0.025	0.020	0.015
*Iridaceae*	*Irisensata* thumb.	0.037	0.063	0.052	0.039
*Gentianaceae*	*Gentiana macrophylla* Pall.	—	—	—	0.005
	*Comastoma pulmonarium* (Turcz.) Toyok.	—	—	0.003	0.002
	*Gentiana scabra* Bunge.	0.002	0.003	0.001	0.003
*Scrophulariaceae*	*Lancea tibetica* Hook. f. et Thoms.	0.018	0.017	0.001	0.029
	*Pedicularis qinghaiensis* T. Yamaz.	—	0.002	0.0145	0.002
*Labiatae*	*Dracocephalum heterophyllum* Benth.	0.022	0.012	0.054	0.049
*Thymelaeaceae*	*Stellera chamaejasme* L.	0.005	—	0.011	—
*Umbelliferae*	*Bupleurum chinensis* DC.	0.012	0.018	0.026	0.008
*Plantaginaceae*	*Plantago asiatica* L.	—	0.004	—	0.012
*Ranunculaceae*	*Aconitum gymnandrum* Maxim.	—	—	—	0.008
*Caryophyllaceae*	*Silene firma Siebold* & Zucc.	0.002	—	0.009	0.004
*Orchidaceae*	*Herminium monorchis* (Linn.) R. Br.	—	—	—	0.001

**Table 2:** The effects of grazing intensity on plant diversity indexes. CK, no grazing; LG, light grazing; MG, moderate grazing; HG, heavy grazing.

Grazing Intensity	Shannon-Wiener Index (*H*’)	Simpson Index (*D*)	Pielou Index (*J*)	Margalef Index (*Dmg*)
CK	2.340 (0.082) ^a^	0.882 (0.014) ^a^	0.870 (0.022) ^a^	2.360 (0.136) ^a^
LG	2.303 (0.118) ^a^	0.880 (0.012) ^a^	0.861 (0.027) ^a^	2.271 (0.163) ^a^
MG	2.423 (0.021) ^a^	0.886 (0.004) ^a^	0.889 (0.019) ^a^	2.382 (0.096) ^a^
HG	2.456 (0.110) ^a^	0.892 (0.016) ^a^	0.890 (0.017) ^a^	2.312 (0.254) ^a^

Note: Mean and SE (standard error). In the same column, significant differences among grazing treatments are shown via different lowercase letters (*p* < 0.05).

**Table 3:** Effects of grazing intensity on the ecosystem function indicators.

Grazing Intensity	Ecosystem Functions Indicators							
	ANPP (g m^−2^)	BGB (g m^−2^)	SOC (g kg^−1^)	TN (g kg^−1^)	AP (mg kg^−1^)	AN (mg kg^−1^)	PN (mg g^−1^)	PP (mg g^−1^)
CK	323.63 (34.31) ^a^	1185.08 (181.18) ^a^	29.19 (1.12) ^a^	2.98 (0.10) ^a^	16.34 (3.12) ^a^	20.26 (0.95) ^a^	18.80 (0.52) ^a^	1.25 (0.07) ^ab^
LG	633.07 (72.09) ^a^	1111.56 (178.41) ^a^	28.01 (1.42) ^a^	2.85 (0.09) ^a^	21.56 (4.42) ^a^	12.03 (2.09) ^b^	19.96 (0.66) ^a^	1.37 (0.07) ^a^
MG	574.93 (110.17) ^a^	1228.22 (189.37) ^a^	27.92 (1.92) ^a^	2.86 (0.23) ^a^	14.88 (2.80) ^a^	14.73 (0.45) ^ab^	17.05 (0.88) ^ab^	1.15 (0.03) ^ab^
HG	453.59 (87.34) ^a^	829.26 (150.06) ^a^	31.97 (2.47) ^a^	3.22 (0.18) ^a^	13.58 (3.72) ^a^	13.29 (0.86) ^b^	15.02 (0.64) ^b^	1.07 (0.02) ^b^

Note: Mean and SE (standard error); In the same column, significant differences among grazing treatments are shown via different lowercase letters (*p* < 0.05). ANPP, aboveground net primary productivity; BGB, belowground biomass; SOC, soil organic carbon; TN, total nitrogen; AP, soil available phosphorus; AN, soil available nitrogen; PN, plant leaf nitrogen content; PP, plant leaf phosphorus content. CK, no grazing; LG, light grazing; MG, moderate grazing; HG, heavy grazing.

**Table 4:** The information on all ecosystem function indicators and their importance.

Ecosystem Functional Indicators	Importance
Aboveground net primary productivity	Primary production function, a key ecosystem process that supports ecosystem belowground functionality.
Belowground biomass	
Soil organic carbon	Soil carbon sequestration function, and build-up of nutrient pools for plants and microorganisms.
Soil total nitrogen	
Soil available phosphorus	Nutrient cycling function. Soil available phosphorus and soil available nitrogen are important nutrients sources for both microorganisms and plants.
Soil available nitrogen	
Plant nitrogen content	Sustain human welfare, plant nitrogen and phosphorus content embody the nutrient utilization of plants, and involve the chemical cycling of nutrients in ecosystems.
Plant phosphorus content	

## Data Availability

All of the data provided in this study are available within this article.
